# A comparison of oncologic and functional outcomes in patients with pt3a renal cell carcinoma treated with partial and radical nephrectomy

**DOI:** 10.1590/S1677-5538.IBJU.2020.0149

**Published:** 2021-02-28

**Authors:** Ricardo Alvim, Amy Tin, Lucas Nogueira, Souhil Lebdai, Nathan Wong, Toshikazu Takeda, Melissa Assel, A. Ari Hakimi, Karim Touijer, Paul Russo, Jonathan Coleman

**Affiliations:** 1 Memorial Sloan Kettering Cancer Center Department of Surgery NY USA Department of Surgery, Urology Service, Memorial Sloan Kettering Cancer Center, NY, USA; 2 Memorial Sloan Kettering Cancer Center Department of Epidemiology and Biostatistics NY USA Department of Epidemiology and Biostatistics, Memorial Sloan Kettering Cancer Center, NY, USA

**Keywords:** Kidney Neoplasms, Carcinoma, Renal Cell, Nephrectomy

## Abstract

**Hypothesis:**

Partial Nephrectomy is oncological safe in patients with pT3a RCC.

**Purpose:**

To compare the oncological and functional outcomes of patients with pT3a RCC scheduled for PN and RN.

**Materials and Methods:**

We retrospectively reviewed patients with pT3a N0 M0 RCC who underwent partial or radical nephrectomy from 2005 to 2016. Perioperative characteristics, including estimated glomerular filtration rate, tumor size, pathological histology, and RENAL nephrometry score, were compared between patients scheduled for partial or radical nephrectomy. We used multivariable Cox proportional hazards regression models to compare overall survival, cancer-specific survival, and recurrence-free survival between planned procedure type.

**Results:**

Of the 589 patients, 369 (63%) and 220 (37%) were scheduled for radical and partial nephrectomy, respectively; 26 (12%) of the scheduled partial nephrectomy cases were intraoperatively converted to radical nephrectomy. After adjusting for tumor size and histology, there were no statistically significant differences in overall survival (hazard ratio 0.66; 95% CI, 0.38–1.13), cancer-specific survival (hazard ratio 0.53; 95% CI, 0.16–1.75), or recurrence-free survival (hazard ratio 0.66; 95% CI, 0.34–1.28) between patients scheduled for partial or radical nephrectomy. Fewer patients scheduled for partial nephrectomy had estimated glomerular filtration rate reductions 3 to 9 months after surgery than patients scheduled for radical nephrectomy.

**Conclusion:**

We found no evidence that patients scheduled to undergo partial nephrectomy had poorer oncologic outcomes than patients scheduled to undergo radical nephrectomy. In select patients with pT3a renal cell carcinoma in whom partial nephrectomy is deemed feasible by the surgeon, partial nephrectomy should not be discouraged.

## INTRODUCTION

According to European Association of Urology and American Urological Association guidelines, partial nephrectomy (PN) is the standard treatment for T1 renal cell carcinoma (RCC) (1, 2). Compared with radical nephrectomy (RN), PN provides similar oncological control and better preserves renal function (3). PN also reduces cardiovascular morbidity and mortality and improves overall survival, especially in patients with preexisting kidney disease (4, 5). These advantages have led to the broader use of PN in patients with larger, more complex, and more aggressive tumors (6).

Because patients with advanced tumors are often in poor health, PN may improve survival and quality of life over RN. Further, a recent retrospective study demonstrated that nephrectomy-induced chronic renal insufficiency is a risk factor for death by any cause in patients with cT1b renal masses (3). However, only one study has evaluated outcomes after PN in patients with high-risk RCC (more advanced than T1), but it did not stratify patients according to tumor stage (7). Thus, whether the benefits of renal function preservation outweigh the potential risk of local recurrence and progression in patients with higher stage tumors remains controversial. The aim of this study was to compare the oncological and functional outcomes of patients with pT3a RCC scheduled for PN and RN.

## MATERIALS AND METHODS

After receiving institutional review board approval (16-747), we retrospectively reviewed records to identify patients with stage pT3a RCC who underwent PN or RN from 2005 to 2016 at Memorial Sloan Kettering Cancer Center. Patients were excluded from analysis if they had positive lymph nodes at diagnosis (n=2), metastasis at diagnosis (n=16), solitary kidney (n=2), hereditary cancer syndrome (n=1), benign lesions (n=19), histology other than conventional/clear cell, chromophobe, or papillary (n=49), or missing data (n=12).

Comparisons of patient and perioperative characteristics between groups were made using Wilcoxon rank-sum for continuous variables and Fisher's exact test for categorical variables. Outcomes included estimated blood loss, warm ischemia time, estimated glomerular filtration rate (eGFR) preoperatively and 6 months postoperatively, length of hospital stay, lymph node invasion (LNI), margin status, Fuhrman grade (clear cell or papillary histology only), tumor size, pathological histology, symptoms at presentation (symptomatic of RCC [flank pain, hematuria, flank mass, and weight loss] or incidental) and RENAL nephrometry score (8). RENAL scores were categorized as low (scores 4 to 6), moderate (scores 7 to 9), or high (score >9). We calculated eGFR using the Chronic Kidney Disease Epidemiology Collaboration (CKD-EPI) formula using serum creatinine levels prior to surgery and 6 months (within 3 to 9 months) after surgery.

We used multivariable Cox proportional hazards regression models, adjusting for LNI, symptoms at presentation (incidental vs. symptomatic), histology (clear cell vs. chromophobe vs. papillary), and tumor size, to assess whether planned surgery type is associated with differences in overall survival (OS), cancer-specific survival (CSS), or recurrence-free survival (RFS). As a sensitivity analysis, we excluded patients with tumor sizes larger than 7cm, for whom it could be argued that they would rarely be treated with PN. All statistical analyses were conducted using STATA 13.0 (StataCorp, College Station, TX).

## RESULTS

We identified 589 patients treated surgically for stage T3a RCC between 2005 and 2016. Of these, 369 (63%) and 220 (37%) were scheduled for RN and PN, respectively. Twenty-six (12%; 95% confidence interval [CI], 8-17) patients who were originally scheduled for PN were converted to RN intraoperatively. Of the 589 patients, 107 patients died, 31 from RCC. The median follow-up time for survivors was 2.0 years after surgery; 71 patients developed a recurrence, and 77 patients were followed for five years without an event.

Table-1 compares the perioperative characteristics of patients scheduled to undergo PN and RN. The overall rate of major complications was similar between scheduled PN and RN (5.0% vs. 3.3%, difference 1.7%, 95% CI, −1.7-5.0; p=0.3); three of the complications were urinary leaks. There were no significant differences in blood loss or length of hospital stay. Based on tumor characteristics, patients scheduled to undergo PN were at lower risk of cancer progression than those scheduled to undergo RN. Patients in the scheduled PN group had smaller tumors by pathology than patients scheduled to undergo RN (4cm vs. 8cm; p <0.0001). A smaller proportion of patients scheduled to undergo PN had conventional clear cell histology on pathology than patients scheduled for RN (77% vs. 88%; p=0.002). Among patients with clear cell or papillary histology, a larger proportion of patients scheduled for PN had cancers with a Fuhrman grade of 1 or 2 on pathology than patients scheduled for RN (25% vs. 11.3%; p <0.0001). Patients in the scheduled PN group had fewer complex tumors based on RENAL score (18% vs. 2.7%; p <0.0001; Table-2).

**Table 1:** Demographic, clinical, and perioperative characteristics of patients with pT3a RCC (n=589) categorized according to planned procedure. All values are median (interquartile range) or frequency (percentage).

		Radical nephrectomy n=369 (63%)	Partial nephrectomy 1 n=220 (37%)	p-value
Age at surgery (years)		62 (53-69)	63 (55-71)	0.3
Male		259 (70%)	157 (71%)	0.8
**Race**				0.3
	White	315 (85%)	187 (85%)	
	Black	5 (1.4%)	7 (3.2%)	
	Asian	24 (6.5%)	10 (4.5%)	
	Other	5 (1.4%)	2 (0.9%)	
	Unknown	20 (5.4%)	14 (6.4%)	
Incidental symptoms at presentation		208 (56%)	186 (85%)	<0.0001
**Pathological histology**				0.002
	Clear cell	323 (88%)	170 (77%)	
	Papillary	13 (3.5%)	21 (10%)	
	Chromophobe	33 (8.9%)	29 (13%)	
**Fuhrman grade (FG)**^2^				<0.0001
	FG 1	1 (0.3%)	0 (0%)	
	FG 2	35 (10%)	47 (25%)	
	FG 3	172 (51%)	104 (54%)	
	FG 4	115 (34%)	23 (12%)	
	Not reported	13 (3.9%)	17 (8.9%)	
High pathologic grade (3/4; N=497)		287 (89%)	127 (73%)	<0.0001
Tumor size (cm)		8 (6-11)	4 (3-5)	<0.0001
Lymph node dissection		286 (78%)	44 (20%)	<0.0001
Lymph node invasion		34 (12%)	0 (0%)	0.013
Positive surgical margin		12 (3.3%)	19 (8.6%)	0.007
Length of hospital stay (days; N=553)		2 (2-3)	3 (2-4)	0.3
Estimated blood loss (mL)		300 (150-500)	300 (150-500)	>0.9

1 Includes 26 patients who were converted to radical nephrectomy intraoperatively.

2 Excludes patients with chromophobe histology.

**Table 2:** RENAL nephrometry scores and pre- and postoperative renal function of patients with stage pT3a RCC (n=589) categorized according to planned procedure. Values are the median (interquartile range) estimated glomerular filtration rate in mL/min/1.73m^2^ or the number of patients (percentage). Normal estimated glomerular filtration values are X-YmL/min/1.73m^2^. eGFR, estimated glomerular filtration rate.

		Radical nephrectomy n=369 (63%)	Partial nephrectomy^1^ n=220 (37%)	p-value
**Complexity based on RENAL score**				<0.0001
	Low	10 (2.7%)	39 (18%)	
	Moderate	87 (24%)	109 (50%)	
	High	216 (59%)	49 (22%)	
	Unknown	56 (15%)	23 (10%)	
	Preoperative eGFR	70.4 (58.1-85.8)	71.1 (57.1-84.7)	0.7
	Preoperative eGFR >60	269 (73%)	158 (72%)	0.8
	6-month postoperative eGFR (N=472)	51.7 (43.8-63.8)	64.2 (47.4-79.1)	<0.0001
**6-month postoperative eGFR >60**		92 (25%)	106 (48%)	<0.0001
	Unknown	76 (21%)	41 (19%)	
**6-month postoperative eGFR lower than baseline**		281 (76%)	138 (63%)	<0.0001
	Unknown	76 (21%)	41 (19%)	

**eGFR** = estimated glomerular filtration rate

1 Includes 26 patients who were converted to radical nephrectomy intraoperatively.

After adjusting for LNI, symptoms at presentation, tumor size, and histology, we did not observe a significant association between planned PN and OS (hazard ratio [HR] 0.66; 95% CI, 0.38-1.13; p=0.13), CSS (HR 0.53; 95% CI, 0.16-1.75; p=0.3), or RFS (HR 0.66; 95% CI, 0.34-1.28; p=0.2) on multivariable analysis. However, the hazard ratios are indicative of improved survival of patients scheduled for PN and suggest that surgeon bias in selecting PN for patients with lower risk tumors is not adequately accounted for in our confounder adjustment.

Although preoperative renal function was similar between the two groups of patients, PN was associated with better postoperative renal function (Table-2). A smaller proportion of patients scheduled to undergo PN had reduced postoperative eGFR levels relative to patients undergoing RN (63% vs. 76%; 13% difference, 95% CI, 6-21; p <0.0001). Results for all outcomes were similar in our sensitivity analyses that excluded 240 patients with tumors larger than 7cm.

## DISCUSSION

Although PN has become increasingly used to treat higher stage RCC, especially in high-volume experienced centers (6), the safety and benefits of PN for pT3a tumors are underreported (9, 10). We did not find a significant difference in complication rate, OS, CSS, or RFS between planned PN and RN for pT3a RCC. Thus, PN does not appear to put patients with pT3a tumors at higher risk of complications or inferior oncologic outcomes. Although the hazard ratios were suggestive of improved outcomes with PN, this effect is likely due to selection bias, with patients with less aggressive tumors preferentially selected for PN. Yet patients scheduled for PN still had non-marginal rates of adverse histologic characteristics: 77% had clear cell RCC, 73% had high-grade tumors, 22% had a RENAL score ≥9, and at least 25% had tumors >5cm. Although other groups have reported on outcomes of high-stage RCC (11-13), our study is one of the largest that describes the management of higher stage tumors by PN.

In a recent National Cancer Database analysis, Maurice et al. reported higher positive surgical margins (PSM) after PN among patients with >1 adverse pathological feature, including pT3a tumors (6). In our study, the higher rate of PSM in the scheduled PN group was not associated with worse oncologic outcomes, including RFS or CSS. This is consistent with a previous study from our institution showing minimal effect of PSM on the CSS of patients with pT3a RCC (14). Other studies of PN (that primarily included patients with lower stage RCC) similarly found no effect of PSM on CSS (15, 16). This suggests that the increased risk of PSM due to the greater procedural complexity of PN does not appear to compromise oncological outcomes, which is consistent with data demonstrating that protection from recurrence is not ensured by negative surgical margins (17).

One important and expected finding in our study was the improved functional outcomes of patients scheduled for PN, with fewer patients having decreased eGFR levels 6 months after surgery than those who underwent RN (62% vs. 76%; −14% difference, 95% CI, −21 to −6). This finding, particularly in the case of small and low-stage tumors, is corroborated by other studies (18, 19). The improved functional outcomes of patients scheduled for PN cannot be explained by baseline differences, as preoperative eGFR was similar between the two groups. Further, 80% of patients had stage 2 or 3 chronic kidney disease (baseline eGFR 30-89mL/min/1.73m2) prior to surgery, in agreement with the finding by Lowrance et al. that chronic kidney disease is associated with higher RCC risk (20).

There are certain limitations of our study that need to be acknowledged. Longer follow-up may be needed to accurately compare the effects of PN and RN on renal function, as it may take more than 2 years for eGFR to return to baseline for almost 50 percent of patients (21). Furthermore, this was a non-randomized and retrospective study, making it subject to selection bias; patients with more complex and higher risk tumors were more frequently in the planned RN group. This is most apparent in our survival analysis, in which there was improved survival among patients scheduled to undergo PN, suggesting that the bias of choosing PN for lower risk tumors was not fully accounted for in our model. Nearly all studies comparing PN and RN outcomes share this design (9, 11, 13). A randomized prospective study with a longer follow-up is necessary to clarify the potential benefits of PN in high-stage RCC.

## CONCLUSION

We found no evidence that patients with pT3a RCC scheduled to undergo PN had poorer oncologic outcomes than patients scheduled to undergo RN. Additionally, patients in the scheduled PN group had better postoperative renal function than patients scheduled for RN. These findings suggest that PN should not be discouraged in select patients with pT3a RCC in whom PN is deemed feasible by the surgeon.

## ABBREVIATIONS

CI: confidence interval
CKD-EPI: chronic kidney disease epidemiology collaboration
CSS: cancer-specific survival
eGFR: estimated glomerular filtration rate
FG: Fuhrman grade
HR: hazard ratio
LNI: lymph node invasion
OS: overall survival
PN: partial nephrectomy
PSM: positive surgical margins
RCC: renal cell carcinoma
RFS: recurrence-free survival
RN: radical nephrectomy
